# Supplementary material for: Interleukin-4 and -13 Gene Expression Profiles in Immune-Related Bullous Pemphigoid Indicate Efficacy of IL-4/IL-13 Inhibitors
Source: Cancers (Basel). 2025 May 31;17(11):1845. doi: 10.3390/cancers17111845 (PMC12153709; doi:10.3390/cancers17111845)
Supplement: Supplementary file 1 [file cancers-17-01845-s001.zip › cancers-3675318-supplementary.pdf]

cancers

MDPI

Supplementary Table S1: Detailed description of the selected genes relevant in the IL-4 and -13 pathway, including p-values. The expression of *IL-4* and *-13,* their receptor genes, and downstream *genes JAK1, TYK2, STAT 3/6, MAPK 1/3* and *CCL26* were examined. (a) BP vs. healthy skin, (b) irBP vs. healthy skin, (c) irBP vs. BP; *: Significantly changed gene expression. padj: adjusted p-value. BP: Bullous pemphigoid, irBP: Immune-related bullous pemphigoid. ↑: Upregulated Gene Expression; -: No significant changes in Gene Expression.

(a)

**BP vs. healthy skin**							
Symbol	GeneID	Alias	Description	log2FoldChange	pvalue	padj	Gene Expression
*IL13RA2**	3598	*CD213A2|CT19|IL-13R|IL13BP*	interleukin 13 receptor_ alpha 2	3.7625	2.9173e-09	3.9500e-07	↑
IL13RA1	3597	*CD213A1|CT19|IL-13Ra|NR4*	interleukin 13 receptor_ alpha 1	0.4210	2.1464e-05	0.0002306	-
*IL13**	3596	*IL-13|P600*	interleukin 13	2.0733	0.0003644	0.0020058	↑
*IL4R**	3566	*CD124|IL-4RA|IL4RA*	interleukin 4 receptor	1.2330	1.1450e-11	3.8759e-09	↑
*STAT3**	6774	*ADMIO|APRF|HIES*	signal transducer and activator of transcription 3	0.8008	0.0005306	0.0026806	↑
*STAT6**	6778	*D12S1644|IL-4-STAT|STAT6B|STAT6C*	signal transducer and activator of transcription 6_ interleukin-4 induced	1.0345	4.7721e-06	6.7306e-05	↑
JAK1	3716	*JAK1A|JAK1B|JTK3*	Janus kinase 1	0.1503	0.1795	0.2840	-
TYK2	7297	*IMD35|JTK1*	tyrosine kinase 2	0.1195	0.2223	0.3352	-
*IL4**	3565	*BCGF-1|BCGF1|BSF-1|BSF1|IL-4*	interleukin 4	2.0159	0.00024	0.00132	↑
*CCL26**	10344	*IMAC|MIP-4a|MIP-4alpha|SCYA26|TSC-1*	chemokine ligand 26	3.6872	0.000142	0.000972	↑
ERK1 (MAPK3)	5595	*ERK-1|ERK1|ERT2|HS44KDAP*	mitogen-activated protein kinase 3	0.5431	0.00017	0.00109	-
ERK2 (MAPK1)	5594	*ERK|ERK-2|ERK2|ERT1|MAPK2*	mitogen-activated protein kinase 1	-0.0111	0.9379	0.9520	-

(b)

**irBP vs. healthy skin**							
Symbol	GeneID	Alias	Description	log2FoldChange	pvalue	padj	Gene Expression
*IL13RA2**	3598	*CD213A2|CT19|IL-13R|IL13BP*	interleukin 13 receptor_ alpha 2	3.4206	1.5945e-09	5.7067e-08	↑
IL13RA1	3597	*CD213A1|CT19|IL-13Ra|NR4*	interleukin 13 receptor_ alpha 1	0.2617	0.0082361	0.0170	-

*IL13**	3596	*IL-13*|*P600*	interleukin 13	2.8558	1.5015e-05	6.9459e-05	↑
IL4R	3566	*CD124*|*IL-4RA*|*IL4RA*	interleukin 4 receptor	1.1055	7.5885e-11	4.6911e-09	-
STAT3	6774	*ADMIO*|*APRF*|*HIES*	signal transducer and activator of transcription 3 (acute-phase response factor)	0.8877	5.5512e-07	5.4272e-06	-
STAT6	6778	*D12S1644*|*IL-4-STAT*|*STAT6B*|*STAT6C*	signal transducer and activator of transcription 6_ interleukin-4 induced	1.1724	5.7336e-12	6.4981e-10	-
JAK1	3716	*JAK1A*|*JAK1B*|*JTK3*	Janus kinase 1	-0.0836	0.4161	0.4896	-
TYK2	7297	*IMD35*|*JTK1*	tyrosine kinase 2	0.0915	0.1913	0.2551	-
*IL4**	3565	*BCGF-1*|*BCGF1*|*BSF-1*|*BSF1*|*IL-4*	interleukin 4	2.7050	1.6504e-06	1.1854e-05	↑
*CCL26**	10344	*IMAC*|*MIP-4a*|*MIP-4alpha*|*SCYA26*|*TSC-1*	chemokine ligand 26	4.3974	1.5799e-06	1.1552e-05	↑
ERK1 (MAPK3)	5595	*ERK-1*|*ERK1*|*ERT2*|*HS44KDAP*	mitogen-activated protein kinase 3	0.50618	4.49679	0.00017	-
ERK2 (MAPK1)	5594	*ERK*|*ERK-2*|*ERK2*|*ERT1*|*MAPK2*	mitogen-activated protein kinase 1	-0.0462	0.7325	0.7758	-

(c)

**irBP vs. BP**							
Symbol	GeneID	Alias	Description	log2FoldChange	pvalue	padj	Gene Expression
IL13RA2	3598	*CD213A2*|*CT19*|*IL-13R*|*IL13BP*	interleukin 13 receptor_ alpha 2	0.2887	0.6076	0.8215	-
IL13RA1	3597	*CD213A1*|*CT19*|*IL-13Ra*|*NR4*	interleukin 13 receptor_ alpha 1	0.0623	0.4631	0.7452	-
IL13	3596	*IL-13*|*P600*	interleukin 13	-0.9297	0.1787	0.4645	-
IL4R	3566	*CD124*|*IL-4RA*|*IL4RA*	interleukin 4 receptor	0.02319	0.8808	0.9536	-
STAT3	6774	*ADMIO*|*APRF*|*HIES*	signal transducer and activator of transcription 3 (acute-phase response factor)	-0.2074	0.2732	0.5833	-
STAT6	6778	*D12S1644*|*IL-4-STAT*|*STAT6B*|*STAT6C*	signal transducer and activator of transcription 6_ interleukin-4 induced	-0.2841	0.1627	0.4461	-
JAK1	3716	*JAK1A*|*JAK1B*|*JTK3*	Janus kinase 1	0.1198	0.3564	0.6649	-
TYK2	7297	*IMD35*|*JTK1*	tyrosine kinase 2	-0.0631	0.5428	0.7987	-
IL4	3565	*BCGF-1*|*BCGF1*|*BSF-1*|*BSF1*|*IL-4*	interleukin 4	-0.5813	0.3189	0.6265	-
CCL26	10344	*IMAC*|*MIP-4a*|*MIP-4alpha*|*SCYA26*|*TSC-1*	chemokine ligand 26	-0.1992	0.7593	0.8786	-

ERK1 (MAPK3)	5595	*ERK-1|ERK1|ERT2|HS44KDAP*	mitogen-activated protein kinase 3	-0.0789	0.6080	0.8215	-
ERK2 (MAPK1)	5594	*ERK|ERK-2|ERK2|ERT1|MAPK2*	mitogen-activated protein kinase 1	-0.0461	0.7953	0.9042	-

Supplementary Table S2: All 168 DEGs of BP vs. healthy skin organized by expression difference (biggest difference 1) in n=17 BP vs. n=24 healthy skin. Genes involved in the IL-4 and IL-13 pathway are bolded. (a) 150 upregulated absolute fold change descending (b) 18 downregulated absolute fold change descending. BP: Bullous pemphigoid. DEG: Differentially expressed genes.

(a) 150 Upregulated absolute fold change descending: In n=17 BP vs. n=24 healthy skin

1	***IL13RA2***	31	*CD180*	61	*CARD9*	91	*BST2*	121	*DDX58*
2	***CCL26***	32	*CLEC4A*	62	*FCER1G*	92	***IL4R***	122	*CD3EAP*
3	*CCL1*	33	*MX1*	63	*IFIT1*	93	*ITGA4*	123	*NOD1*
4	*S100A12*	34	*PDCD1*	64	*F12*	94	*IL1RAP*	124	*MASP1*
5	*GZMB*	35	*CCL13*	65	*IL6R*	95	*IL1R1*	125	*CDK1*
6	*S100A8*	36	*ULBP2*	66	*CEACAM6*	96	*IFITM2*	126	*IFIH1*
7	*LILRA5*	37	*IRF7*	67	*OAS3*	97	*NCF4*	127	*PVR*
8	*MEFV*	38	*CD163*	68	*IL1R2*	98	*ANXA1*	128	*MAP2K2*
9	*IL1RL1*	39	*HLA-DRB3*	69	*CTSL*	99	*SH2B2*	129	*RELB*
10	*TNFRSF11B*	40	*VEGFA*	70	*IRF4*	100	*TNFSF10*	130	*NOD2*
11	*CCR7*	41	*THY1*	71	*RUNX1*	101	*CCR2*	131	*THBD*
12	*ISG15*	42	*TNFRSF4*	72	*TAPBP*	102	*ATG12*	132	*MAF*
13	*LBP*	43	*CD68*	73	*CD63*	103	*CCR1*	133	*IL18R1*
14	*IL1RN*	44	*TNFSF13*	74	*HLA-C*	104	*TAP2*	134	*GPI*
15	*CCL23*	45	*CSF2RB*	75	*CD14*	105	*CSF1R*	135	***STAT2***
16	*CD209*	46	*CD24*	76	*IRF8*	106	*IKBKB*	136	***STAT3***
17	*LILRB3*	47	*C4B*	77	*IL12RB1*	107	*NFATC4*	137	*TNFRSF11A*
18	*POU2AF1*	48	*FCER2*	78	*LAIR2*	108	*AXL*	138	*ELK1*
19	*S100A7*	49	*CCL19*	79	*AMICA1*	109	*NFKB2*	139	*MAPKAPK2*
20	*FCGR2A*	50	*CLEC7A*	80	*ISG20*	110	*PRG2*	140	*HLA-A*
21	*IFIT2*	51	*NT5E*	81	*IFI16*	111	***STAT6***	141	*ITGA5*
22	*CR1*	52	*SBNO2*	82	*C3AR1*	112	*PSMB10*	142	*BAX*
23	*CCL18*	53	*TLR9*	83	*FCGR3A*	113	*CD74*	143	*MIF*
24	*THBS1*	54	*C1QB*	84	***JAK3***	114	*CD44*	144	*CD276*
25	*TNFRSF12A*	55	*FCGR1A*	85	*NRP1*	115	*TICAM1*	145	*LY86*
26	***IL4***	56	*SIGLEC1*	86	*CD33*	116	*IFNAR2*	146	*ITGA6*
27	*SELE*	57	*C2*	87	*BTK*	117	*MYD88*	147	*IKBKG*
28	*PLAU*	58	*CD274*	88	*MRC1*	118	*CD4*	148	*RELA*
29	***IL13***	59	*CSF3R*	89	*CCND3*	119	*LTBR*	149	*EWSR1*
30	*CD1D*	60	*TGFB1*	90	*IL3RA*	120	*ENG*	150	*BCL10*

(b) 18 Downregulated absolute fold change descending: In n=17 BP vs. n=24 healthy skin

1	*FOS*	5	*MUC1*	9	*NCAM1*	13	*ALCAM*	17	*ABCB1*
2	*HSD11B1*	6	*TMEFF2*	10	*APOE*	14	*CD200*	18	*CD164*
3	*CLEC5A*	7	*RORC*	11	*IL1RAPL2*	15	*GATA3*		
4	*LRRN3*	8	*EGR2*	12	*ARG2*	16	*TLR5*		

Supplementary Table S3: All 99 DEGs of irBP vs. healthy skin organized by expression difference (biggest difference 1) in n=19 irBP vs. n=24 healthy skin. Genes involved in the IL-4 and IL-13 pathway are bolded. (a) 82 upregulated absolute fold change descending (b) 17 downregulated absolute fold change descending. irBP: Immune-related bullous pemphigoid. DEG: Differentially expressed genes.

(a) 82 Upregulated absolute fold change descending: In n=19 irBP vs. n=24 healthy skin

1	***CCL26***	18	*TNFRSF11B*	35	*CCL13*	52	*FCER2*	69	*CD274*
2	*S100A12*	19	*CCL23*	36	*SELE*	53	*CTLA4*	70	*IL3RA*
3	*GZMB*	20	*CD1D*	37	*IL2RA*	54	*IRF8*	71	*GNLY*
4	*CCL1*	21	*POU2AF1*	38	*TNFRSF4*	55	*CCL19*	72	*CD8B*
5	*CXCL6*	22	*CXCL13*	39	*CR1*	56	*CD24*	73	*CD86*
6	*S100A8*	23	*MS4A1*	40	*FCGR3A*	57	*C2*	74	*SLAMF1*
7	***IL13RA2***	24	*CD209*	41	*TNFRSF12A*	58	*CD8A*	75	*TGFB1*
8	*LILRA5*	25	*LBP*	42	*CD163*	59	*CCL18*	76	*CLEC7A*
9	*MEFV*	26	*CXCL11*	43	*IRF7*	60	*CLEC4A*	77	*THBS1*
10	*PDCD1*	27	*FCGR2A*	44	*HLA-DRB3*	61	*LAG3*	78	*C4B*
11	*CXCL1*	28	*IL1RN*	45	*CD68*	62	*C3AR1*	79	*IL12RB1*
12	*CCR7*	29	*CEACAM6*	46	*C1QB*	63	*FCER1G*	80	*CTSL*
13	*CXCL9*	30	*ULBP2*	47	*FUT7*	64	*IRF4*	81	*IL6R*
14	***IL13***	31	*FCGR1A*	48	*PLAU*	65	*CD37*	82	*CXCR3*
15	*CXCL10*	32	*CSF2RB*	49	*ISG15*	66	*TNFRSF17*		
16	***IL4***	33	*TNFSF18*	50	*MX1*	67	*HLA-C*		
17	*LILRB3*	34	*CD180*	51	*TLR9*	68	*THY1*		

(b) 17 Downregulated absolute fold change descending: In n=19 irBP vs. n=24 healthy skin

1	*TNFSF11*	5	*MBL2*	9	*BAGE*	13	*NEFL*	17	*CTAG1B*
2	*FOS*	6	*SYCP1*	10	*CLEC5A*	14	*ROPN1*		
3	*KIR3DL3*	7	*FUT5*	11	*TPTE*	15	*CT45A1*		
4	*CCL25*	8	*CTCFL*	12	*IL1RAPL2*	16	*HSD11B1*		

Supplementary Table S4: 13 DEGs of irBP vs. BP organized by expression difference (biggest difference 1) in n=19 irBP vs. n=17 BP. (a) 4 upregulated absolute fold change descending (b) 9 downregulated absolute fold change descending. irBP: Immune-related bullous pemphigoid. DEG: Differentially expressed genes.

(a) 4 Upregulated absolute fold change descending: In n=19 irBP vs. n=17BP

1	*TNFSF11*	2	*CCL25*	3	*MBL2*	4	*TPTE*

(b) 9 Downregulated absolute fold change descending: In n=19 irBP vs. n=17BP

1	*CXCL9*	3	*CXCR5*	5	*APOE*	7	*CD200*	9	*NLRC5*
2	*CXCL13*	4	*CD8A*	6	*IL16*	8	*ADA*		
